# PARP inhibitor exerts an anti-tumor effect via LMO2 and synergizes with cisplatin in natural killer/T cell lymphoma

**DOI:** 10.1186/s12916-023-02904-9

**Published:** 2023-07-13

**Authors:** Jiazhuo Wu, Cunzhen Shi, Hongwen Li, Wenting Song, Shuo Huang, Jianxiang Zhang, Wencai Li, Zhaoming Li, Mingzhi Zhang

**Affiliations:** 1 Department of Oncology, The First Affiliated Hospital of Zhengzhou University, Zhengzhou, 450052 Henan China; 2 Department of Infectious Diseases and Hepatology, The First Affiliated Hospital of Zhengzhou University, Zhengzhou, 450052 Henan China; 3 Department of Pathology, The First Affiliated Hospital of Zhengzhou University, Zhengzhou, 450052 Henan China

**Keywords:** PARP inhibitor, LMO2, Natural killer/T cell lymphoma, DNA damage repair

## Abstract

**Background:**

PARP inhibitor (PARPi), as a kind of DNA damage repair inhibitor, has been shown to be effective in various solid tumors and hematologic malignancies. Natural killer/T cell lymphoma (NKTCL) is a highly aggressive malignancy, the treatment of which has long been a major challenge in the clinic. Here, we investigated the efficacy and mechanism of PARPi, and the therapeutic value of PARPi combined with cisplatin in NKTCL.

**Methods:**

The cell proliferation, cell apoptosis, and cell cycle of NKTCL cells were detected respectively by CCK-8 and flow cytometry. The changes of mRNA expression and protein level were measured respectively by mRNA-sequencing, quantitative real-time PCR, western blotting, and immunofluorescence. LMO2 expression was detected by immunohistochemistry and western blotting. Targeted knockdown of LMO2 was conducted by short hairpin RNA. The tumor xenograft models were established to evaluate the efficacy of drugs in vivo*.*

**Results:**

PARPi inhibited cell proliferation, promoted cell apoptosis, and induced S-phase cell cycle arrest in NKTCL cells. PARPi led to the accumulation of DNA damage by blocking DNA repair and DNA replication. Additionally, LMO2 deficiency reduced the sensitivity of NKTCL cells to PARPi. Finally, the combination of PARPi and cisplatin exhibited significant synergistic effects both in vitro and in vivo.

**Conclusions:**

In summary, we found that PARPi exerted an anti-tumor effect via LMO2 and synergized with cisplatin in NKTCL, which provides the theoretical basis for the clinical application of PARPi.

**Supplementary Information:**

The online version contains supplementary material available at 10.1186/s12916-023-02904-9.

## Background

PARP inhibitor (PARPi), as a kind of DNA damage repair inhibitor, mainly prevents DNA single-strand breaks (SSBs) repair, and unrepaired SSBs transform into more injurious but less repairable DNA double-strand breaks (DSBs) [1]. In rapidly proliferating tumor cells, there are various mutations or deletions in the DNA damage response (DDR) pathway, which results in DSBs being unable to be repaired and tumor cell death. Previously, the most classic finding was that tumor cells with BRCA1/2 dysfunction were highly sensitive to PARPi alone, namely, “synthetic lethality” [2, 3]. It has been known that BRCA1/2 was critical to repair DSBs by homologous recombination [4]. Therefore, PARPi initially was approved for solid tumors with BRCA1/2 dysfunction, such as ovarian, breast, and prostate cancers, instead of hematologic malignancies lacking BRCA1/2 mutation [5–8].

Recently, it has been confirmed that PARPi was available in the treatment of hematologic malignancies with other DDR dysfunctions. It was worth noting that diffuse large B cell lymphoma (DLBCL), T cell acute lymphoblastic leukemia (T-ALL), and follicular lymphoma cells with high LMO2 expression were sensitive to PARPi, where LMO2 interacting with 53BP1 inhibited the DSBs repair [9]. Besides, high LMO2 expression was associated with favorable prognosis in patients with different hematologic malignancies including B cell acute lymphoblastic leukemia (B-ALL), T lymphoblastic leukemia/lymphoma (T-LL), and DLCBL [10–14]. In brief, LMO2, expressed in all tissues except mature T cells, could predict sensitivity to PARPi and patients’ prognosis in different hematologic malignancies.

Natural killer/T cell lymphoma (NKTCL) is a type of highly aggressive non-Hodgkin’s lymphoma with an extremely poor prognosis. For advanced-stage patients, the mainly suggested options are combination chemotherapy and chemoradiation therapy, such as DDGP, P-GEMOX, and radiotherapy combined with DeVIC [15–17]. Platinum-based chemotherapeutic drugs, including cisplatin, carboplatin, and oxaliplatin, as one of the most classic chemotherapy agents in the treatment of various tumors, had been included in the above-mentioned regimens. It is well known that platinum-based chemotherapeutic drugs represented by cisplatin exert an anti-tumor effect by forming platinum–DNA adduct, blocking DNA replication, inhibiting DNA damage, and inducing cell apoptosis [18, 19].

However, for advanced-stage NKTCL patients, the 5-year progression-free survival (PFS) rate and overall survival (OS) rate were still less than 20% and 50%, respectively [20–22]. It is necessary to develop novel therapeutic agents to improve the prognosis. Here, we investigated whether PARPi exerted an anti-tumor effect via LMO2 in NKTCL cells, and based on this, we further explored the therapeutic value of PARPi combined with cisplatin.

## Methods

### Reagents

Olaparib and niraparib were purchased from Selleckchem (China). Fluzoparib was a gift from Jiangsu Hengrui Pharmaceuticals Co., Ltd. (Jiangsu, China). Pamiparib was a gift from Beigene (Beijing) Biotechnology Co., Ltd. (Beijing, China). All of the drugs were dissolved in dimethyl sulfoxide to obtain a stock solution of 10 mM and stored at − 80 °C. Cisplatin was obtained from Jiangsu Hausen Pharmaceutical Co., Ltd. (Jiangsu, China) and stored at 5 mg/mL at room temperature (RT). D-Luciferin (sodium salt) was purchased from Glpbiochem (China), dissolved in PBS stored at − 80 °C in the dark.

### Cell lines and culture

NKYS, KHYG1, YT, and NKL were cultured in RPMI 1640 medium (Gibco, USA) supplemented with 10% fetal bovine serum (Clack, USA) and 1% penicillin/streptomycin (Invitrogen, California, USA). Additionally, NKYS, KHYG1, and NKL were recombinant human interleukin-2-dependent (100 IU/mL, PeproTech, Rocky Hill, NJ, USA). SNK6 was cultured in X-VIVO medium (Lonza, USA) with 500 IU/mL recombinant human interleukin-2. NKYS, KHYG1, and YT were kindly obtained from Dr. Wing C. Chan (City of Hope Medical Center). SNK6 was kindly provided by Dr. Norio Shimizu and Yu Zhang of Chiba University, and NKL was purchased from the cell bank of the Bena Culture Collection (Beijing, China).

### Cell proliferation, cell apoptosis, and cell cycle

A total of 2 × 10^3^ cells were seeded into 96-well plates and treated with different concentrations of different agents. After 72 h, CCK-8 solution (US Everbright Inc., Jiangsu, China) was added to each well and allowed to react for another 1 h at 37 °C. Then, the absorbance value at optical density (OD) 450 nm was measured by a Multiskan FC microplate reader (Thermo Scientific, Waltham, MA, USA). The half maximal inhibitory concentration (IC_50_) values were predicted using SPSS software version 25.0 (IBM Corp.). The synergistic effects were estimated by combination index (CI) value using the Chou–Talalay method and CompuSyn software (CompuSyn Inc.). CI < 1: synergism; CI = 1: additive effect; CI > 1: antagonism. Independent experiments were repeated at least three times.

A total of 2 × 10^5^ cells were seeded in 24-well plates and treated with different concentrations of different agents for 48 h or 72 h. For cell apoptosis analysis, cells were stained with Annexin V-APC and PI/7-AAD (Keygen Biotech, Jiangsu, China) in the dark for 15 min at RT. For cell cycle analysis, cells were fixed in 75% ethanol overnight at 4 °C and then digested with RNase A and PI (Keygen Biotech, Jiangsu, China) in the dark for 30 min at RT. Then, all these cells were analyzed by a FACS Calibur flow cytometer (BD Biosciences, NJ, USA). Independent experiments were repeated at least three times.

### mRNA-seq (mRNA-sequencing) analysis

Total RNA extraction, mRNA library construction, sequencing, and data analysis were performed by Shanghai Yuanqi Biomedical Technology Co., Ltd. (Shanghai, China) according to the standard procedure. Brief procedures are listed in Additional file 1. The raw sequencing data are available from the NCBI and are archived under accession number PRJNA884169.

### Quantitative real-time PCR (qRT-PCR)

Total RNA was extracted using TRIzol reagent (Invitrogen, Carlsbad, CA, USA) from NKTCL cells according to the manufacturer’s instructions. The concentration and purity of these samples were measured by Nanodrop 1000 spectrophotometry (Thermo Scientific, Wilmington, DE, USA). cDNA was synthesized by reverse transcription using UEIris RT mix with DNase (US Everbright Inc., Jiangsu, China), and qRT-PCR was performed using Universal SYBR Green qPCR Supermix (US Everbright Inc., Jiangsu, China). The primers were synthesized by Hangzhou Shangyasai Biotechnology Co., Ltd. (Hangzhou, China), and the sequences are listed in Additional file 2: Table S1. GAPDH was used as a reference gene for mRNA quantitation. The relative expression level was calculated with the 2^−ΔΔCt^ method.

### Western blotting

Cells were lysed in cold RIPA lysis buffer with protease and phosphatase inhibitor cocktail (Thermo Scientific, Waltham, MA, USA) for 30 min on ice. The cell lysates were clarified by centrifugation at 13,000 × g for 30 min. Proteins were resolved on gels with different concentrations by SDS-PAGE and transferred onto PVDF membranes (Amersham Biosciences, Piscataway, NJ, USA). The membranes were blocked in TBST buffer containing 5% non-fat milk for 1 h at RT. Then, the membranes were incubated with primary antibodies overnight at 4 °C and secondary antibodies for 1 h at RT. The antibodies are listed in Additional file 2: Table S2. The band images were digitally captured and quantified with a ChemiDoc™ XRS + system (Bio-Rad Laboratories, Hercules, CA, USA).

### Immunofluorescence

Cells were washed with PBS, fixed with 4% formaldehyde for 15 min at RT, and permeabilized with 0.5% Triton X-100 for another 15 min at RT. The cells were incubated with primary antibodies at 4 °C overnight and secondary antibodies in the dark for 1 h at RT. Then, the cells were stained with DAPI (US Everbright Inc, Jiangsu, China) in the dark for 5 min. Images were captured using a Zeiss Axio Imager M2 microscope (Carl Zeiss Corporation, Germany). The antibodies are listed in Additional file 2: Table S2.

### Clinical samples and immunohistochemistry (IHC)

In this study, we collected 67 tumor tissues from NKTCL patients who were histologically and clinically diagnosed by the department of pathology and oncology between 2014 and 2019 at the First Affiliated Hospital of Zhengzhou University. The IHC protocol was performed as the standard streptavidin–biotin-peroxidase-immunostaining procedure. The antibodies are listed in Additional file 2: Table S2. We defined that LMO2 expression was scored as negative or positive based on a 30% cut-off, which is based on previous studies in DLBCL and T-ALL [12, 13]. The scores were assessed by pathologists without prior knowledge of the patients’ information. Moreover, the detailed clinical characteristics of these patients are listed in Additional file 2: Table S3. The protocols were approved by the Institutional Research Ethics Committee of the First Affiliated Hospital of Zhengzhou University (Lot No.2022-KY-1061–002).

### Construction of stable cell lines

Stable LMO2 knockdown cell lines were generated using lentiviral constructs expressing short hairpin RNA (shRNA) of LMO2 (shLMO2, target sequence: AGGTGACAGATACCTCCTCAT) and negative control (shNC, target sequence: TTCTCCGAACGTGTCACGT). Stable 53BP1 knockdown cell lines were generated using lentiviral constructs expressing shRNA of 53BP1 (sh53BP1, target sequence: GATACTGCCTCATCACAGT) and negative control (shNC, target sequence: TTCTCCGAACGTGTCACGT). All of these were designed and provided by Shanghai Genechem Co., Ltd. (Shanghai, China).

A total of 2 × 10^5^ cells were seeded in 24-well plates, which were infected with 10μL HitransG P solution and lentivirus including shNC, shLMO2, or sh53BP1 (MOI = 1:10). After more than 48 h, the cells were transferred to a fresh complete medium, which included 1 mg/mL puromycin. Subsequently, the transfection efficiency was measured by flow cytometry and western blotting, and stable cell lines were established for the following experiments.

### Co-immunoprecipitation (Co-IP)

Cells were lysed in cold IP lysis buffer with protease and phosphatase inhibitor cocktail (Thermo Scientific, Waltham, MA, USA) for 30 min on ice. Cell debris was removed by centrifugation at 13,000 × g for 30 min. Cell lysate (1 mg) was combined with antibody and incubated overnight at 4 °C with rotation. The antigen–antibody mixture was added to the tube containing prewashed agarose beads and incubated at RT for 1 h with mixing. After washing five times with IP lysis buffer, the antigen–antibody complex was eluted before SDS-PAGE. The antibodies are listed in Additional file 2: Table S2.

### In vivo therapy on NKTCL-cell-drived xenograft models

Twenty NSG mice (3–4 weeks old, female, 15–20 g) were purchased from the Shanghai Model Organisms Center (Shanghai, China). A total of 1 × 10^7^ NKYS-Luciferase cells dissolved in a mixture of 200 µL medium and 200 µL matrigel (Corning Incorporated, USA) were injected into the right axillary region by subcutaneous inoculation to establish the tumor xenograft models. After one week, these mice were randomly divided into four groups (*n* = 5) and given different treatments by intraperitoneal injections (i.p.): control, fluzoparib (40 mg/kg, twice every 5 days), cisplatin (2 mg/kg, three times in the first week), and fluzoparib (40 mg/kg, twice every 5 days) plus cisplatin (2 mg/kg, three times in the first week).

Tumor size was measured every week, and tumor volume (V) was calculated using the following formula: V = ab^2^/2 (a: the long diameter and b: the short diameter). Mice were given D-Luciferin (150 mg/kg, i.p.) and anesthetized with isoflurane. After 5 min, luminescence was detected using the in vivo imaging system (IVIS), and the intensity was quantitated and normalized by Living Image software (PerkinElmer, Massachusetts, USA).

The protocol was approved by the Institutional Research Ethics Committee of the First Affiliated Hospital of Zhengzhou University (Lot No.2022-KY-1061–002).

### Hematoxylin–eosin (HE) staining

First, we executed mice using the cervical dislocation method on day 49 after tumor cells inoculation. The fresh tissues (hearts, livers, kidneys, and lungs) were collected from mice, then put into 4% paraformaldehyde, embedded in paraffin, and cut into 5 μm-thick sections. The next procedures were as follows: coating, dewaxing, dehydration, hematoxylin, differentiation, bluing, eosin, dehydration, clearing, and cover-slipping. Two senior pathologists viewed cellular and tissue structure using a Zeiss Axio Imager M2 microscope (Carl Zeiss Corporation, Germany).

### NK cell isolation and culture

First, peripheral blood mononuclear cells (PBMCs) were obtained by Ficoll-Paque density gradient centrifugation from the blood of healthy donors. NK cells were isolated from PBMCs using CD56 microbeads (Miltenyi Biotec, Germany). CD3 − CD56 + NK cells (more than 95%) were selected and cultured in RPMI 1640 with 15% fetal bovine serum and 100 IU/mL interleukin-2 for the next experiments.

### Statistical analysis

Statistical analyses were performed using GraphPad Prism version 8.0 (GraphPad Software, Inc., La Jolla, CA, USA). Data are expressed as the mean ± standard deviation (SD). Comparisons between groups were performed using Student’s *t* test and analysis of variance (ANOVA), respectively. PFS and OS were calculated using the Kaplan–Meier method and log-rank test. The correlation between LMO2 expression and clinicopathologic features was assessed using *χ*^2^-test. A value of *p* < 0.05 was considered statistically significant.

## Results

### PARPi exert an anti-tumor effect in NKTCL cells both in vitro and in vivo

**Fig. 1:**
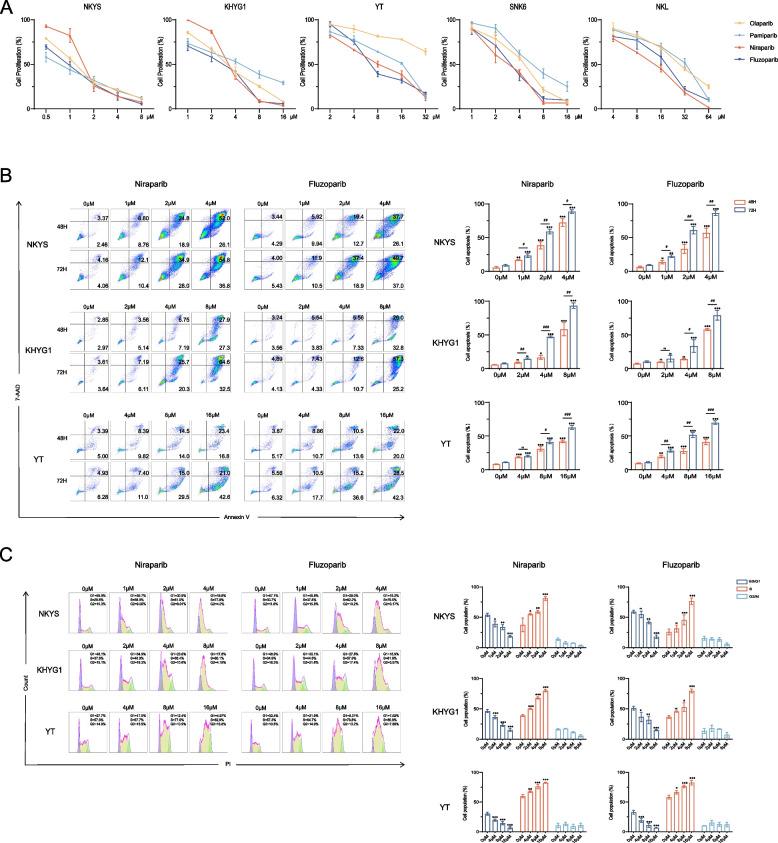
PARPi exert an anti-tumor effect in vitro by inhibiting cell proliferation, promoting cell apoptosis, and inducing S-phase cell cycle arrest in NKTCL. **A** CCK-8 assays in NKTCL cells treated with different concentrations of olaparib, pamiparib, niraparib, and fluzoparib at 72 h. **B** Apoptosis assays in NKTCL cells treated with different concentrations of niraparib and fluzoparib at 48 h or 72 h. The percentage of apoptotic cells was measured by flow cytometry. **C** Cell cycle assays in NKTCL cells treated with different concentrations of niraparib and fluzoparib at 72 h. The percentage of cell cycle distribution at the G0/G1, S, and G2/M phases was measured by flow cytometry. All data are expressed as the mean ± standard deviation (SD). NS, not significant. ^*^P < 0.05, ^**^P < 0.01, ^***^P < 0.001 vs. the control group, ^#^*P* < 0.05, ^##^*P* < 0.01, ^###^*P* < 0.001 vs. 48 h group

To investigate the effect of PARPi on NKTCL cell proliferation, NKTCL cells were treated with PARPi at different concentrations for 72 h, and CCK-8 reagent was added to measure OD values at 450 nm. PARPi significantly inhibited the proliferation of NKTCL cells in a dose-dependent manner (Fig. 1A), and NKTCL cells were more sensitive to niraparib (IC_50_ values for NKYS, KHYG1, YT, SNK6, and NKL were respectively 1.45 μM, 3.43 μM, 7.15 μM, 3.00 μM, and 11.64 μM) and fluzoparib (IC_50_ values for NKYS, KHYG1, YT, SNK6, and NKL were respectively 0.88 μM, 2.22 μM, 5.68 μM, 3.40 μM, and 17.59 μM). Next, for the following experiments, we explored the cell apoptosis and cell cycle of NKTCL cells after treatment with fluzoparib or niraparib. NKYS, KHYG1, and YT cells were treated with fluzoparib and niraparib at different concentrations for 48 h or 72 h, and cell apoptosis and the cell cycle were analyzed by flow cytometry. Apoptotic cells increased in a dose- and time-dependent manner (Fig. 1B). In addition, the number of cells in the S phase obviously increased, while the percentage of cells in the G0/G1 phase decreased as the concentration increased (Fig. 1C). All of these results suggested that PARPi showed an anti-tumor effect to some extent in vitro, and this effect was progressively enhanced with increasing concentration and duration. Moreover, primary human NK cells, as a negative control cell sample, were treated with PARPi at different concentrations for 72 h, and CCK-8 reagent was added to measure OD values at 450 nm. It was found that there was no obvious change in the survival rates (more than 80%) of NK cells on concentrations effective for NKTCL cells (Additional file 3: Fig. S1).

Consequently, we established NKTCL-cell-drived NSG mouse xenograft models to explore the anti-tumor effect of fluzoparib in vivo. During the treatment of fluzoparib (40 mg/kg, twice every 5 days), tumor volume and fluorescence gradually decreased in the fluzoparib group while gradually increased in the control group (Additional file 3: Fig. S2). After six weeks of treatment, we executed mice using the cervical dislocation method and performed the HE staining on the hearts, livers, kidneys, and lungs of mice to evaluate the toxicity of fluzoparib in vivo*.* There was no obvious histological change in these tissues, which suggested that fluzoparib could be administered without related toxicity in mice (Additional file 3: Fig. S3).

### Fluzoparib leads to the accumulation of DNA damage by blocking DDR and DNA replication

**Fig. 2:**
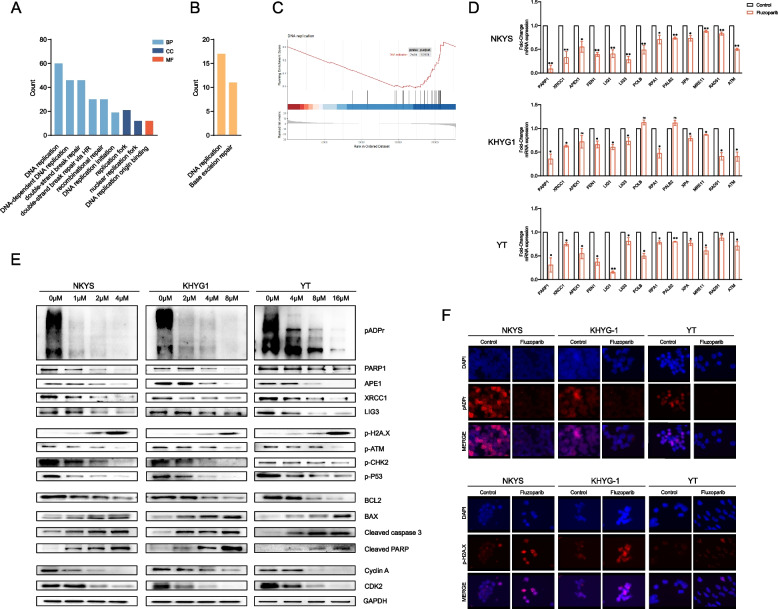
Fluzoparib leads to the accumulation of DNA damage by blocking DDR and DNA replication. **A**–**C** The results of mRNA-seq were analyzed by GO network (**A**), KEGG network (**B**), and GSEA (**C**) analyses. **D** qRT-PCR for DDR-related mRNAs. **E** Western blotting for DDR-related, cell apoptosis-related, and cell cycle-related proteins. **F** Immunofluorescence for pADPr and p-H2A.X level. All data are expressed as the mean ± SD. NS, not significant. ^*^P < 0.05, ^**^P < 0.01, ^***^P < 0.001 vs. the control group

Subsequently, we performed mRNA-seq of the NKYS cell line to analyze the differentially expressed genes (DEGs) after treatment with fluzoparib. The DEGs were enriched in pathways related to DNA replication, DNA repair, and DDR (*p* < 0.05) by GO network, KEGG network, and GSEA analyses (Fig. 2A–C). Furthermore, we verified a number of related molecules in the DDR by qRT-PCR and western blotting. On the one hand, there was a noticeable reduction in the expression of several essential molecules in the DDR, such as PARP1, APEX1, LIG1, XRCC1, RPA1, ATM, and RAD51 (Fig. 2D, E). On the other hand, the expression of proteins associated with cell apoptosis and the cell cycle was correspondingly altered upon treatment with increasing concentrations of fluzoparib (Fig. 2E). We found that poly-ADP-ribose (pADPr) was downregulated and p-H2A.X was upregulated after treatment with fluzoparib, as shown by western blotting and immunofluorescence (Fig. 2E, F).

### LMO2 deficiency reduces the sensitivity of NKTCL cells to fluzoparib

**Fig. 3:**
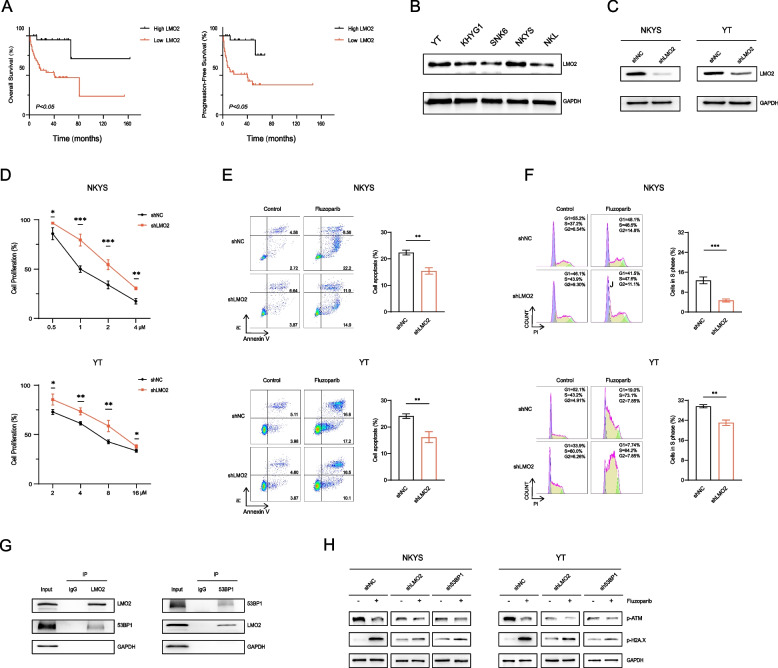
LMO2 deficiency reduces the sensitivity of NKTCL cells to fluzoparib. **A** Kaplan–Meier curves for PFS and OS in 67 NKTCL patients according to the LMO2 level. *P* values from log-rank test. **B** Western blotting for LMO2 in NKTCL cell lines. **C** Western blotting for LMO2 in NKYS and YT cells infected with shNC and shLMO2. **D** Cell proliferation curves for shNC and shLMO2 cells treated with fluzoparib for 72 h. **E** Apoptosis assays in shNC and shLMO2 cells treated with fluzoparib. The difference in the apoptotic rate before and after treatment was calculated. The difference value between shNC cells and shLMO2 cells was compared. **F** Cell cycle assays in shNC and shLMO2 cells treated with fluzoparib. The difference in the S-phase rate before and after treatment was calculated. The difference value between shNC cells and shLMO2 cells was compared. **G** Co-immunoprecipitation for the interaction of LMO2 and 53BP1 in NKYS cells. **H** Western blotting for p-ATM and p-H2A.X levels in shNC, shLMO2, and sh53BP1 cells treated with fluzoparib. All data are expressed as the mean ± SD. NS, not significant. ^*^P < 0.05, ^**^P < 0.01, ^***^P < 0.001 vs. the shNC group

First, we evaluated the expression of LMO2 in tumor tissues from 67 NKTCL patients by immunohistochemistry. Based on a 30% cut-off, there were 22 cases for positive LMO2 expression and 45 cases for negative LMO2 expression (Additional file 3: Fig. S4). The results of Kaplan–Meier survival analysis showed that NKTCL patients with positive LMO2 expression had better PFS and OS (Fig. 3A). We analyzed the relationship between LMO2 expression and clinicopathological features of these patients, and the results showed that LMO2 expression had a significant correlation with the stage of disease (*p* = 0.022), which revealed that LMO2 expression may be associated with NKTCL progression (Additional file 2: Table S3). In addition, we examined the expression of LMO2 in different NKTCL cell lines (Fig. 3B).

According to the results, we constructed stable LMO2-downregulated NKYS and YT cell lines (Fig. 3C). Compared to shNC cells, the ability of fluzoparib to inhibit cell proliferation, promote cell apoptosis, and induce S-phase cell cycle arrest was weakened in shLMO2 cells (Fig. 3D–F). The apoptosis rate (%) increased from 7.30 to 30.76 in NKYS-shNC cells and from 10.51 to 26.80 in NKYS-shLMO2 cells. The apoptosis rate (%) increased from 9.09 to 33.80 in YT-shNC cells and from 8.47 to 26.60 in YT-shLMO2 cells. The proportion of S phase (%) increased from 37.20 to 46.50 in NKYS-shNC cells and from 43.90 to 47.60 in NKYS-shLMO2 cells. The proportion of S phase (%) increased from 43.20 to 73.10 in YT-shNC cells and from 60.00 to 84.20 in YT-shLMO2 cells. In brief, the absence of LMO2 reduced the sensitivity of NKTCL cells to fluzoparib.

The previous studies have confirmed that LMO2 inhibited DSBs repair by interacting with 53BP1, and 53BP1 could promote ATM-mediated signaling as a key member in DDR [9, 23]. To further explore the mechanism by which LMO2 affects the sensitivity of NKTCL cells to fluzoparib, we examined the interaction of LMO2 with 53BP1 by Co-IP in the NKYS cell line, and the result showed that LMO2 formed a complex with 53BP1 (Fig. 3G). Next, we constructed stable 53BP1-downregulated NKYS and YT cell lines (Additional file 4: Fig. S5). It was evident that the changes in the expression of p-ATM and p-H2A.X in shLMO2 or sh53BP1 cells were not as apparent as those in shNC cells after the same treatment with fluzoparib (Fig. 3H).

### Fluzoparib combined with cisplatin shows synergistic effects both in vitro and in vivo

**Fig. 4:**
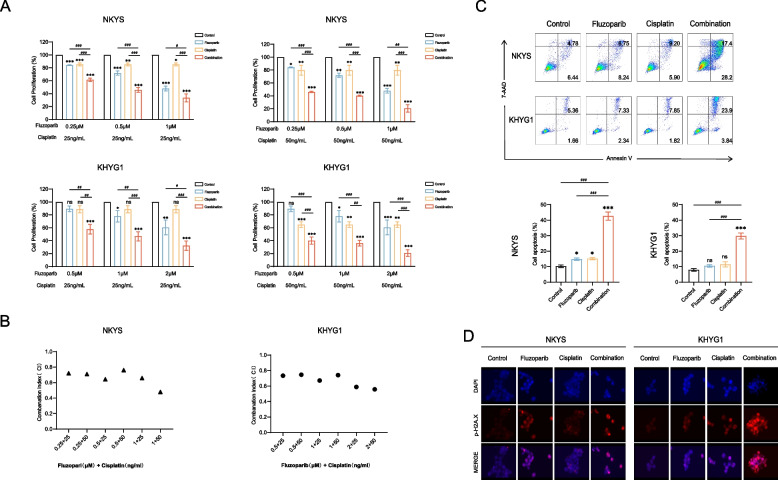
Fluzoparib combined with cisplatin shows synergistic effects in vitro*.* **A** Cell proliferation curves in NKYS and KHYG1 cells treated with different combinations of fluzoparib and cisplatin at 72 h. **B** CI values for the results shown in **A**. **C** Apoptosis assays in NKYS and KHYG1 cells were divided into the control group, fluzoparib group, cisplatin group, and combination group. **D** Immunofluorescence for the p-H2A.X level in the four groups. All data are expressed as the mean ± SD. NS, not significant. ^*^P < 0.05, ^**^P < 0.01, ^***^P < 0.001 vs. the control group, ^#^*P* < 0.05, ^##^*P* < 0.01, ^###^*P* < 0.001 vs. fluzoparib or cisplatin group

To explore the anti-tumor effect of fluzoparib combined with cisplatin, we detected the proliferation of NKTCL cells treated with different combinations of drugs. The results showed that, compared to fluzoparib or cisplatin alone, the combinations of the two drugs could more significantly inhibit cell proliferation (Fig. 4A, B). The combination of the two drugs also enhanced the proportion of apoptotic cells and increased the expression of p-H2A.X, a DNA damage-related indicator (Fig. 4C, D). The combined treatment increased the apoptosis rate (%) from 11.22 to 45.60 in NKYS, and 7.02 to 27.74 in KHYG1, which augmented the cell apoptosis more efficiently than fluzoparib or cisplatin alone.

**Fig. 5:**
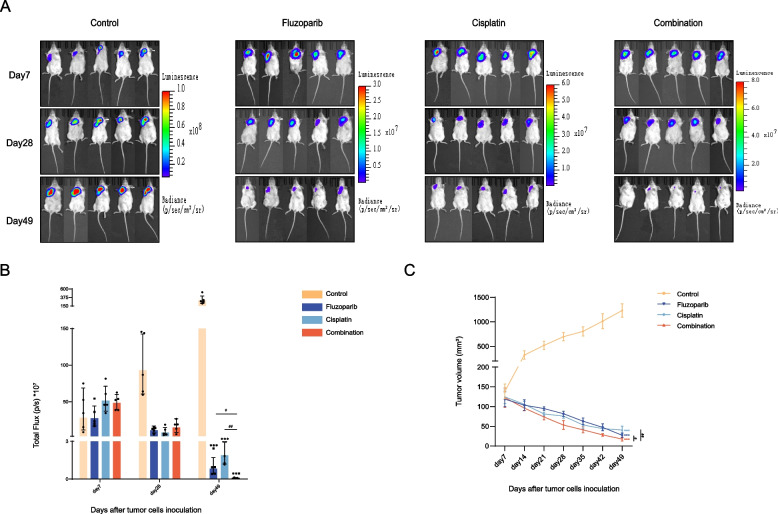
Fluzoparib combined with cisplatin inhibits NKTCL cell growth in vivo*.* **A** IVIS imaging for the tumor burden on days 7, 28, and 49 in the control group, fluzoparib group, cisplatin group, and combination group. **B** Total flux (p/s) *10^7^ values for the result shown in **A**. **C** Tumor volume (mm^3^) curves during treatment. All data are expressed as the mean ± SD. NS, not significant. ^*^P < 0.05, ^**^P < 0.01, ^***^P < 0.001 vs. the control group, ^#^*P* < 0.05, ^##^*P* < 0.01, ^###^*P* < 0.001 vs. fluzoparib or cisplatin group

To explore the anti-tumor effect of fluzoparib combined with cisplatin in vivo, we first established NKTCL-cell-drived NSG mouse xenograft models, which were divided into four groups according to the different treatments. As the duration of treatment increased, tumor volume and fluorescence gradually decreased in the fluzoparib, cisplatin, and combination group while gradually increased in the control group. Importantly, compared with fluzoparib or cisplatin alone, the combination of the two drugs decreased tumor volume and fluorescence more significantly (Fig. 5A–C).

## Discussion

In this study, we first found that PARPi exerted an anti-tumor effect by inhibiting cell proliferation, promoting cell apoptosis, and inducing S-phase cell cycle arrest in NKTCL cells, while there was no obvious toxicity in normal human NK cells. This finding suggested that PARPi may be an effective and safe therapeutic agent for NKTCL. Thus, we further explored the underlying mechanism to explain by which PARPi sensitized NKTCL cells through a series of experiments.

Here, we found that the pathways related to BER and DNA replication were downregulated. It was consistent with the mechanisms of PARPi in past studies: PARPi not only directly inhibited PARP activity, which played a vital role in the BER pathway, but also trapped PARP-DNA complexes causing failure to continue DNA replication correctly [24–26]. Subsequently, unrepaired SSBs transformed into more injurious but less repairable DSBs. If such damage could be repaired timely, the tumor cells would continue to proliferate rapidly. Therefore, we hypothesized that there existed another action that prevented DSBs from being repaired properly in NKTCL cells.

It has been observed that LMO2-positive tumor cells were sensitive to PARPi in various hematologic malignancies, different from solid tumors in which BRCA1/2 deficiency conferred synthetic lethality to PARPi. Interestingly, our results showed that LMO2 expression may be associated with NKTCL progression and NKCTL patients with high LMO2 expression may have better PFS and OS, which was consistent with the previous studies [10–14]. Besides, LMO2 deficiency weakened the anti-tumor effect of PARPi on NKTCL cells. PARPi inhibited the ATM-mediated signaling pathway, and this inhibitory effect was reduced after the downregulated of 53BP1 or LMO2. Thus, we could preliminarily judge the prognosis of NKTCL patients according to LMO2 expression and determine whether PARPi can be used in subsequent treatment sessions. Importantly, it provides a new perspective on the clinical management of NKTCL patients, and different treatments could be chosen for patients depending on their different DDR capabilities.

Chemotherapy is the cornerstone of treatment for advanced-stage NKTCL patients, and cisplatin, as the most widely used anti-tumor drug, has played an irreplaceable role in various solid tumors and hematologic malignancies [19, 27]. Theoretically, both cisplatin and PARPi could increase DNA damage by directly destroying the structure of DNA and preventing the repair of DNA damage, respectively. We explored that PARPi combined with cisplatin indeed exhibited significant synergistic anti-tumor effects by inhibiting cell proliferation, inducing cell apoptosis, and enhancing the DNA damage in NKTCL cells. Meanwhile, many clinical studies have demonstrated that some patients could benefit from platinum-based chemotherapy combined with PARPi. For example, veliparib combined with cisplatin significantly improved PFS in patients with BRCA-like metastatic triple-negative breast cancer [28]. The combination of veliparib with cisplatin and etoposide showed a signal of efficacy in patients with extensive-stage small-cell lung cancer [29]. Veliparib with carboplatin and paclitaxel resulted in significant and durable improvement in PFS in patients with germline BRCA mutation-associated advanced breast cancer [30]. Thus, our finding provided the theoretical basis for the application of PARPi in combination with cisplatin in the clinical management of NKTCL, which meant promising progress for the combination therapy of precision medicine and traditional chemotherapy.

In a word, we clarified that NKTCL cells were sensitive to PARPi, in which LMO2 played an important role in predicting drug sensitivity and prognosis. Besides, PARPi combined with cisplatin also exerted anti-tumor effects in NKTCL. In future research, we need to further explore the precise molecular mechanisms of LMO2 in NKTCL cells and the value of PARPi alone and combined with chemotherapy in relevant clinical trials, such as the scope of applications, the mode of applications, and the corresponding side effects.

## Conclusions

Taken together, PARPi exerts an anti-tumor effect via LMO2 and synergizes with cisplatin in NKTCL, and LMO2 also could predict the prognosis of patients with NKTCL. These findings provide a novel perspective for the clinical management of NKTCL patients.

## Supplementary Information

**Additional file 1.** Brief procedures of mRNA-sequencing analysis.

**Additional file 2: Table S1.** Primers for quantitative real-time PCR. **Table S2.** Information of antibodies applied in immunohistochemistry, immunofluorescence, co-immunoprecipitation, and western blotting. **Table S3.** The correlation between LMO2 expression and clinicopathologic features of 67 patients with NKTCL.

**Additional file 3: Fig. S1.** The CCK-8 assays for human normal NK cellstreated with different concentrations of PARPi were determined to evaluate the toxicity in vitro. **Fig. S2.** Fluzoparib inhibits NKTCL cell growth in vivo. **Fig. S3.** The hematoxylin-eosin staining for organs of mice treated with fluzoparib or not was determined to evaluate the toxicity of fluzoparib in vivo. **Fig. S4.** Representative imagesby immunohistochemistry of NKTCL tissues.

**Additional file 4: Table S4-S8.** Images of original western blots. **Fig. S5.** Images of shNC and sh53BP1 cells.

## Data Availability

The datasets used and analyzed during the current study are available from the corresponding author on reasonable request.

## Abbreviations

ANOVA: Analysis of variance
CI: Combination index
Co-IP: Co-immunoprecipitation
DDR: DNA damage response
DEGs: Differentially expressed genes
DLBCL: Diffuse large B cell lymphoma
DSBs: DNA double-strand breaks
HE: Hematoxylin-eosin
i.p.: Intraperitoneal injections
IF: Immunofluorescence
IHC: Immunohistochemistry
IVIS: In vivo imaging system
mRNA-seq: MRNA-sequencing
NKTCL: Natural killer/T cell lymphoma
OD: Optical density
OS: Overall survival
pADPr: Poly-ADP-ribose
PARPi: PARP inhibitor
PBMCs: Peripheral blood mononuclear cells
PFS: Progression-free survival
qRT-PCR: Quantitative real-time PCR
RT: Room temperature
SD: Standard deviation
shRNA: Short hairpin RNA
SSBs: DNA single-strand breaks
T-ALL: T cell acute lymphoblastic leukemia
V: Tumor volume
