# Supplementary material for: PARP inhibitor exerts an anti-tumor effect via LMO2 and synergizes with cisplatin in natural killer/T cell lymphoma
Source: BMC Med. 2023 Jul 13;21:253. doi: 10.1186/s12916-023-02904-9 (PMC10347840; doi:10.1186/s12916-023-02904-9)
Supplement: Supplementary file 1 — Additional file 1. Brief procedures of mRNA-sequencing analysis. [file 12916_2023_2904_MOESM1_ESM.pdf]

Methods

mRNA-sequencing analysis

Total RNA was extracted from the indicated cells using Trizol (Invitrogen, Carlsbad, CA, USA) according to the manual instruction. Total RNA was qualified and quantified using a Nano Drop and Agilent 2100 bioanalyzer (Thermo Fisher Scientific, MA, USA).

mRNA Library Construction: Oligo(dT)-attached magnetic beads were used to purified mRNA. Purified mRNA was fragmented into small pieces with fragment buffer at appropriate temperature. Then First-strand cDNA was generated using random hexamer-primed reverse transcription, followed by a second-strand cDNA synthesis. Afterwards, A-Tailing Mix and RNA Index Adapters were added by incubating to end repair. The cDNA fragments obtained from previous step were amplified by PCR, and products were purified by AMPure XP Beads, then dissolved in EB solution. The product was validated on the Agilent Technologies 2100 bioanalyzer for quality control. The double stranded PCR products from previous step were heated denatured and circularized by the splint oligo sequence to get the final library. The single strand circle DNA (ssCir DNA) was formatted as the final library. The final library was amplified with phi29 to make DNA nanoball (DNB) which had more than 300 copies of one molecular, DNBs were loaded into the patterned nanoarray and single end 50 bases reads were generated on BGIseq500 platform (BGI-Shenzhen, China).

The sequencing data was filtered with SOAPnuke (v1.5.2). Clean reads were obtained and stored in FASTQ format. The clean reads were mapped to the reference genome using HISAT2 (v2.0.4). Bowtie2 (v2.2.5) was applied to align the clean reads to the reference coding gene set then expression level of gene was calculated by RSEM (v1.2.12). Differential expression analysis was performed using the DESeq2 (v1.4.5) with Q value ≤ 0.05. To take insight to the change of phenotype, KEGG and GO enrichment analysis of annotated different expressed gene was performed by Phyper based on Hypergeometric test. GSEA (version 2.2.4) and gene set collection of KEGG pathways were used for enrichment analysis.
