# Supplementary material for: PARP inhibitor exerts an anti-tumor effect via LMO2 and synergizes with cisplatin in natural killer/T cell lymphoma
Source: BMC Med. 2023 Jul 13;21:253. doi: 10.1186/s12916-023-02904-9 (PMC10347840; doi:10.1186/s12916-023-02904-9)
Supplement: Supplementary file 2 — Additional file 2: Table S1. Primers for quantitative real-time PCR. Table S2. Information of antibodies applied in immunohistochemistry, immunofluorescence, co-immunoprecipitation, and western blotting. Table S3. The correlation between LMO2 expression and clinicopathologic features of 67 patients with NKTCL. [file 12916_2023_2904_MOESM2_ESM.pdf]

Table S1. Primers for quantitative real-time PCR.

Genes	Sequence of the primer (5' to 3')
PARP1-F	CCACACACAATGCGTATGAC
PARP1-R	CCACAGCAATCTTCGGTTA
XRCC1-F	AACTCGTACCCCAGCCACA
XRCC1-R	AGCACCACTACCACACCCTG
APEX1-F	GATTAGATTGGGTAAAGGAAGAAGC
APEX1-R	AGTATTGATGAGAGAGTCCAGGCA
FEN1-F	TGCTAATGCGACACCTGACTG
FEN1-R	CCCAATACCCCGGATACTCT
DNAligase1-F	CTTTGTGGAGACAGAGGGCG
DNAligase1-R	TCTCGTAGGTGGCATCAACATC
DNAligase3-F	GCTATGAAGGTGGGGGAG
DNAligase3-R	GTGGAGGGTGGCAAGTAAA
POLB-F	TAGCAAAATACCCACACAAA
POLB-R	ATGGACCAATGCCACTAACT
RPA1-F	GGGAATGGGTTCTACTGTTT
RPA1-R	GTACGGATCTGACTTTTGTTG
PALB2-F	GTTCCGTAGATGTGAGTGCC
PALB2-R	TCTGCGAAGTGCCAGGTATA
XPA-F	TAACCAAAACAGAGGCAAAA
XPA-R	ACCCCAAACTTCAAGAGACC
MRE11-F	TACGACTGCGAGTGGACTAT
MRE11-R	CTCTATGCCTGAAAAAATGG
RAD51-F	CTCGCTGATGAGTTTGGTGT
RAD51-R	CAGGGAGAGTCGTAGATTTTGC
ATM-F	ATTACGGGTGTTGAAGGTGT
ATM-R	TCATGGTCCAGTCAAAGAGT
GAPDH-F	*CAGGAGGCATTGCTGATGAT*
GAPDH-R	*GAAGGCTGGGGCTCATTT*

Table S2. Information of antibodies applied in immunohistochemistry (IHC), immunofluorescence (IF), co-immunoprecipitation (Co-IP) and western blotting (WB).

Antibody	Sources	
pADPr, 1:100 (IF), 1:1000 (WB)	Santa Cruz	Cat# sc-56198
p-H2A.X (Ser139) 1:100 (IF), 1:1000 (WB)	CST	Cat# 9718
PARP1, 1:1000 (WB)	Abcam	Cat# ab194586
APEX1, 1:1000 (WB)	Abcam	Cat# ab92744
XRCC1, 1:1000 (WB)	Abcam	Cat# ab134056
LIG3, 1:500 (WB)	Proteintech	Cat# 26583-1-AP
p-ATM (Ser1981), 1:1000 (WB)	CST	Cat# 5883
p-CHK2 (Thr68), 1:1000 (WB)	CST	Cat# 2197
p-P53 (Ser15), 1:1000 (WB)	CST	Cat# 9286
BCL2, 1:2000 (WB)	Proteintech	Cat# 60178-1-Ig
BAX, 1:2000 (WB)	Proteintech	Cat# 60267-1-Ig
Cleaved caspase 3, 1:1000 (WB)	CST	Cat# 9661
Cleaved PARP, 1:1000 (WB)	CST	Cat# 5625
Cyclin A, 1:500 (WB)	Abcam	Cat# ab32498
CDK2, 1:500 (WB)	Proteintech	Cat# 60312-1-Ig
GAPDH, 1:3000 (WB)	Proteintech	Cat# 10494-1-AP
LMO2, 1:500 (WB)	Abcam	Cat# ab183328
LMO2, 1:100 (IHC)	Abcam	Cat# ab91652
LMO2, 5 µg per 1 mg protein (Co-IP)	Santa Cruz	Cat# sc-65736
53BP1, 1:1000 (WB)	Abcam	Cat# ab175933
53BP1, 5 µg per 1 mg protein (Co-IP)	Santa Cruz	Cat# sc-515841
Alexa Fluor 594-AffiniPure Fab Fragment Bovine Anti-Goat IgG, Fc Fragment Specific, 1:200 (IF)	Jackson ImmunoResearch	Cat# 805-587-008
HRP-conjugated Affinipure Goat Anti-Rabbit IgG (H+L), 1:5000 (WB)	Proteintech	Cat No. SA00001-2
HRP-conjugated Affinipure Goat Anti-Mouse IgG (H+L), 1:5000 (WB)	Proteintech	Cat No. SA00001-1

Table S3. The correlation between LMO2 expression and clinicopathologic features of 67 patients with NKTCL.

Variable	N	LMO2＋	LMO2－	*P* value
**Total**	67	22	45	
**Gender**				0.101
Male	54	15	39	
Female	13	7	6	
**Age(year)**				0.550
>60	16	4	12	
≤60	51	18	33	
**Ki-67**				0.253
≥50	58	21	37	
<50	9	1	8	
**EBER**				0.713
Negative	1	0	1	
Positive	66	22	44	
**LDH**				0.991
Normal	29	9	20	
Elevated	38	13	25	
**Stage**				**0.022***
I+II	33	14	19	
III+IV	34	8	26	
**IPI score**				0.781
0-2	46	16	30	
3-5	21	6	15	

EBER, Epstein-Barr virus encoding RNAs; LDH, lactate dehydrogenase; IPI, international prognostic index. *$P < 0.05$.
