# Supplementary material for: PARP inhibitor exerts an anti-tumor effect via LMO2 and synergizes with cisplatin in natural killer/T cell lymphoma
Source: BMC Med. 2023 Jul 13;21:253. doi: 10.1186/s12916-023-02904-9 (PMC10347840; doi:10.1186/s12916-023-02904-9)
Supplement: Supplementary file 3 — Additional file 3: Fig. S1. The CCK-8 assays for human normal NK cellstreated with different concentrations of PARPi were determined to evaluate the toxicity in vitro. Fig. S2. Fluzoparib inhibits NKTCL cell growth in vivo. Fig. S3. The hematoxylin-eosin staining for organs of mice treated with fluzoparib or not was determined to evaluate the toxicity of fluzoparib in vivo. Fig. S4. Representative imagesby immunohistochemistry of NKTCL tissues. [file 12916_2023_2904_MOESM3_ESM.pdf]

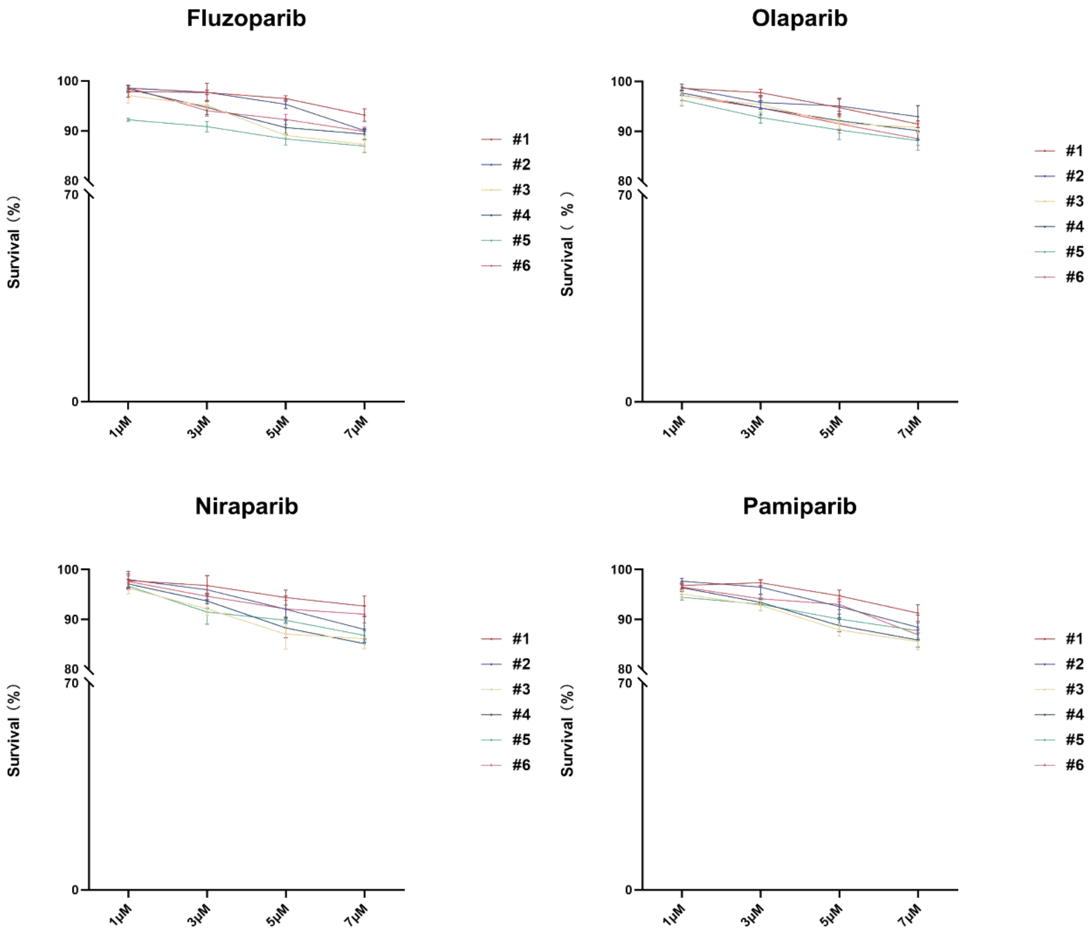

Fig. S1. The CCK-8 assays for human normal NK cells (six cases) treated with different concentrations of PARPis (72 hours) were determined to evaluate the toxicity *in vitro*.

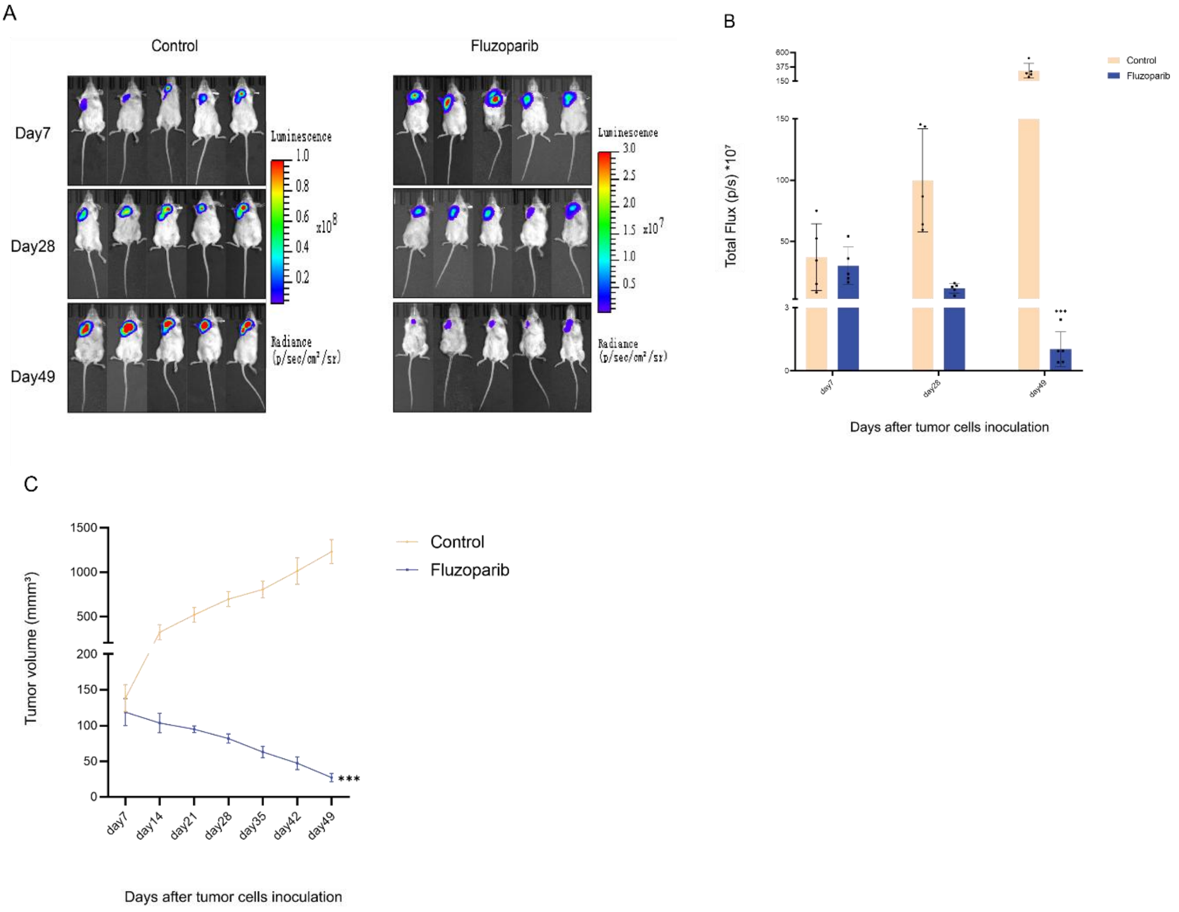

Fig. S2. Fluzoparib inhibits NKTCL cell growth *in vivo*. (A) IVIS imaging for the tumor burden. (B) Total flux (p/s) *10^7 values for the result shown in A. (C) Tumor volume (mmm^3) curves during treatment.

All data are expressed as the mean ± SD.*P < 0.001 vs. the control group.**

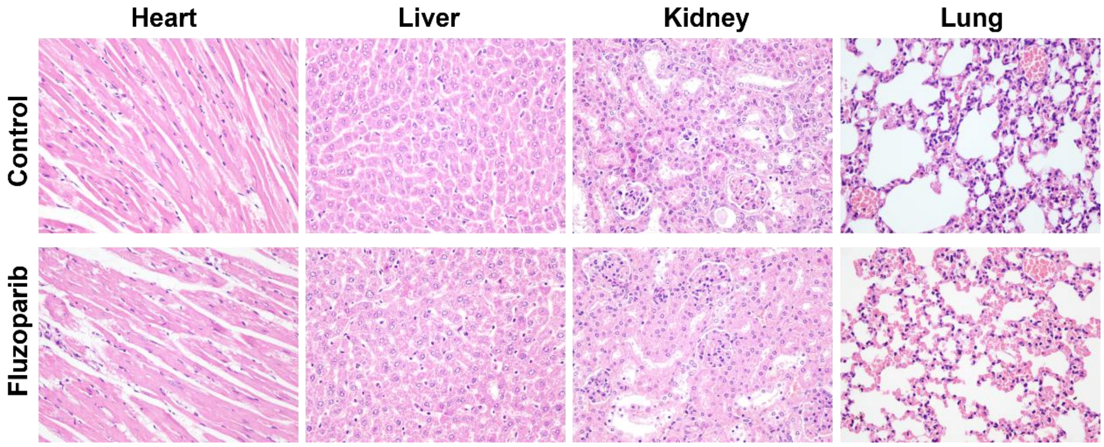

Fig. S3. The hematoxylin-eosin staining (× 100 magnification) for organs (heart, liver, kidney, and lung) of mice treated with fluzoparib or not was determined to evaluate the toxicity of fluzoparib *in vivo*.

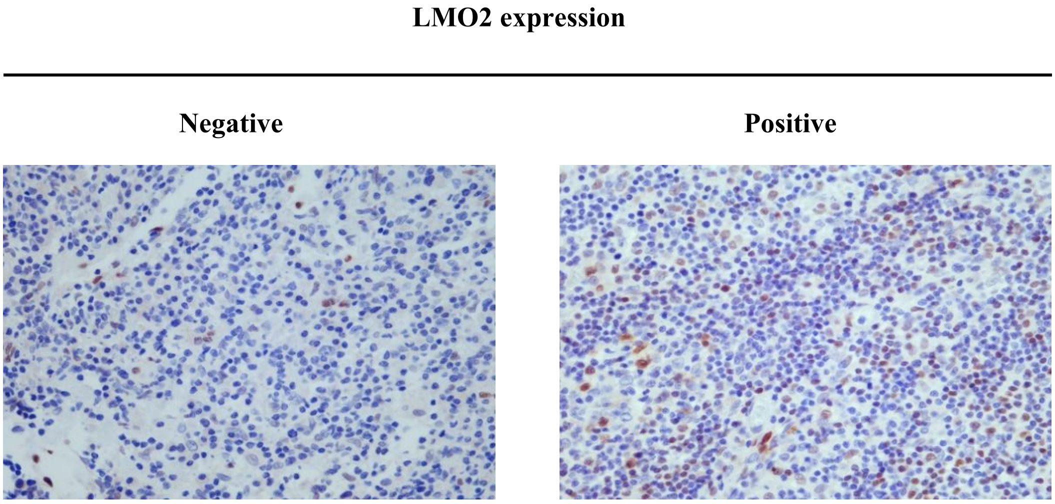

Fig. S4. Representative images (× 400 magnification) by immunohistochemistry of NKTCL tissues.
