# Supplementary material for: PARP inhibitor exerts an anti-tumor effect via LMO2 and synergizes with cisplatin in natural killer/T cell lymphoma
Source: BMC Med. 2023 Jul 13;21:253. doi: 10.1186/s12916-023-02904-9 (PMC10347840; doi:10.1186/s12916-023-02904-9)
Supplement: Supplementary file 4 — Additional file 4: Table S4-S8. Images of original western blots. Fig. S5. Images of shNC and sh53BP1 cells. [file 12916_2023_2904_MOESM4_ESM.pdf]

Table S4. Images in Fig. 2E.

	NKYS	KHYG1	YT
**pADPr**	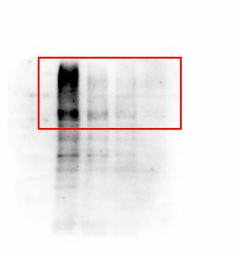	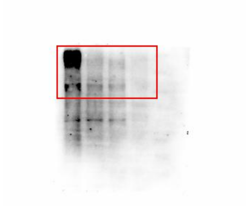	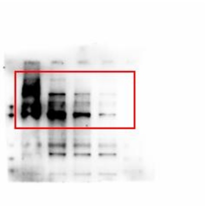
**PARP1**	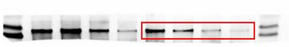	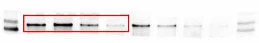	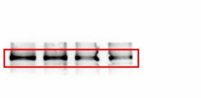
**APE1**	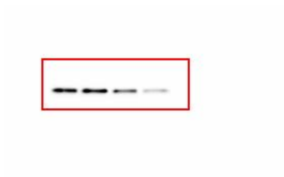	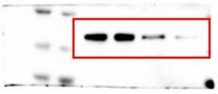	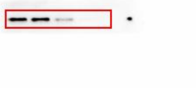
**XRCC1**	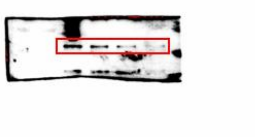	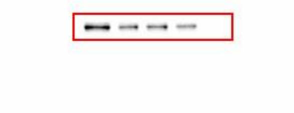	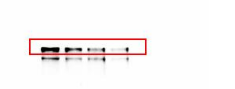
**LIG3**	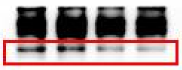	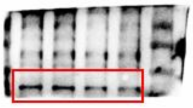	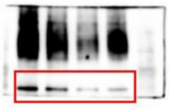
**p-H2AX**	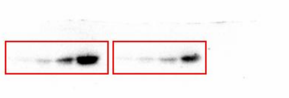	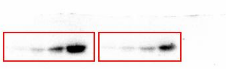	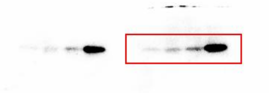
**p-ATM**	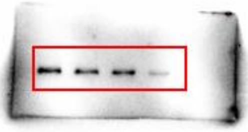	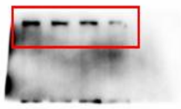	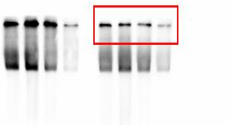
**p-CHK2**	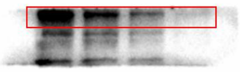	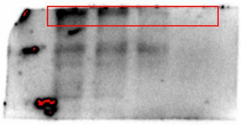	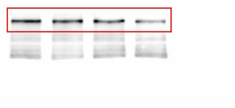

**p-P53**	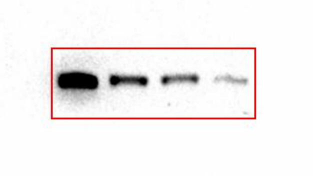	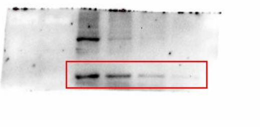	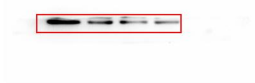
**BCL2**	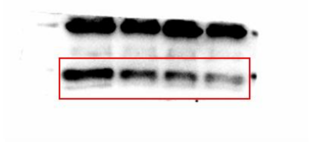	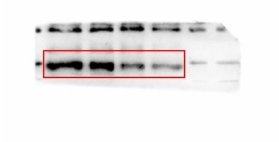	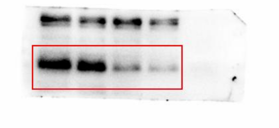
**BAX**	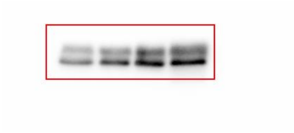	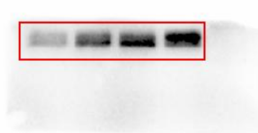	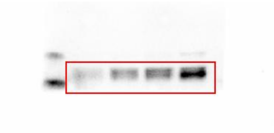
**Cleaved Caspase 3**	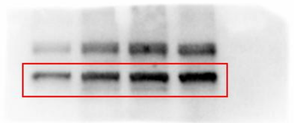	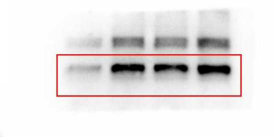	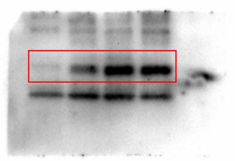
**Cleaved PARP**	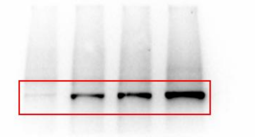	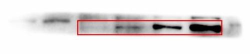	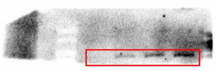
**Cyclin A**	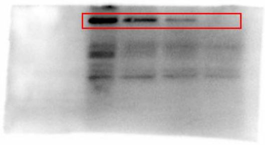	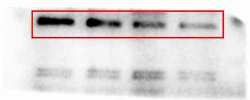	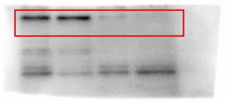
**CDK2**	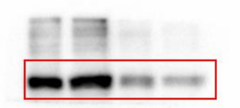	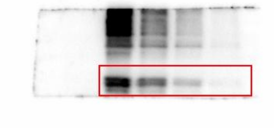	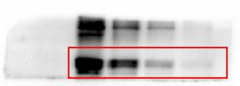
**GAPDH**	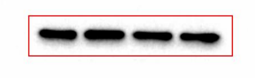	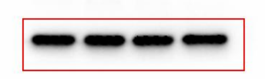	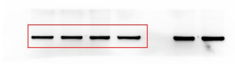

Table S5. Images in Fig. 3B.

**LMO2**	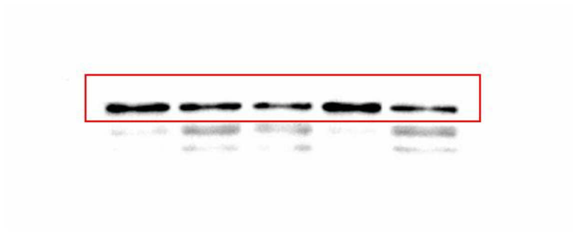
**GAPDH**	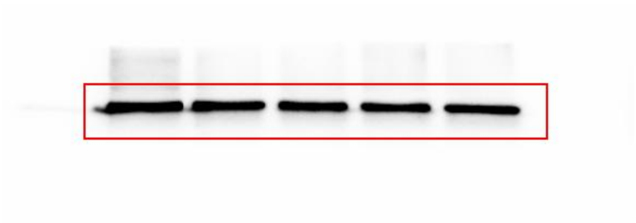

Table S6. Images in Fig. 3C.

	NKYS	YT
**LMO2**	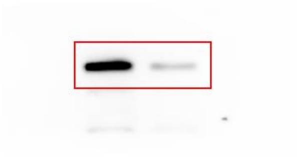	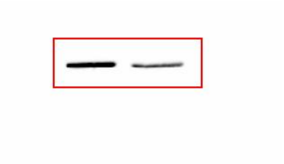
**GAPDH**	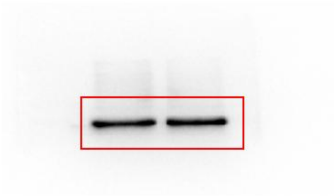	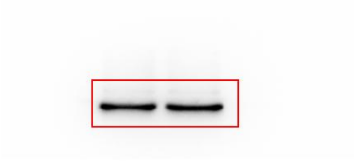

Table S7. Images in Fig. 3G.

**53BP1**	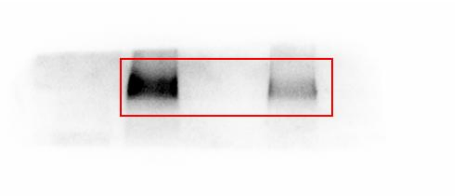	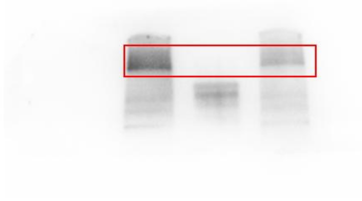
**LMO2**	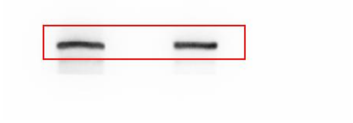	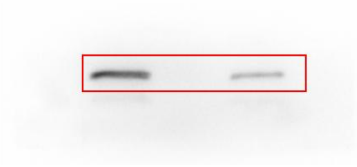
**GAPDH**	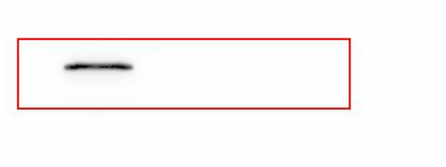	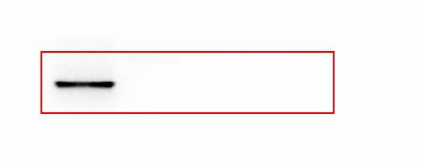

Table S8. Images in Fig. 3H.

	NKYS-shNC	NKYS-shLMO2	NKYS-sh53BP1
p-ATM	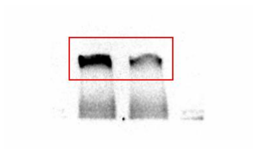	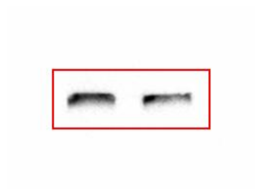	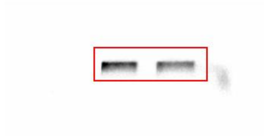
p-H2AX	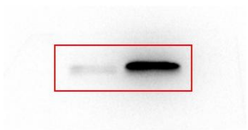	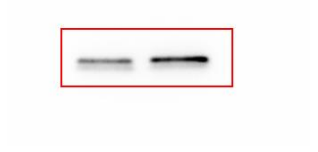	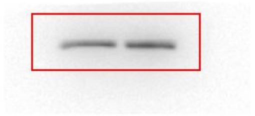
GAPDH	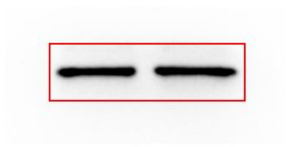	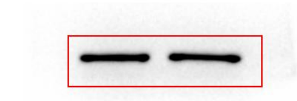	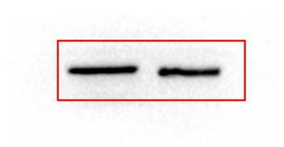

	YT-shNC	YT-shLMO2	YT-sh53BP1
p-ATM	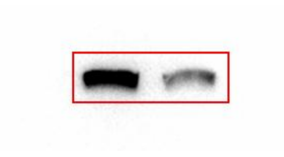	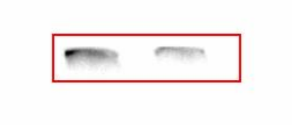	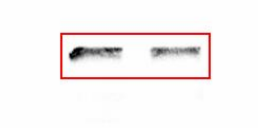
p-H2AX	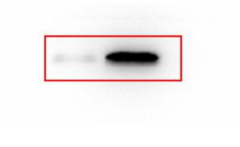	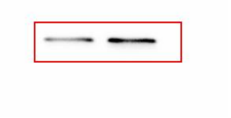	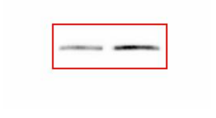
GAPDH	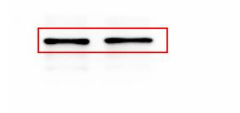	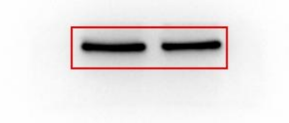	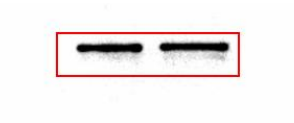

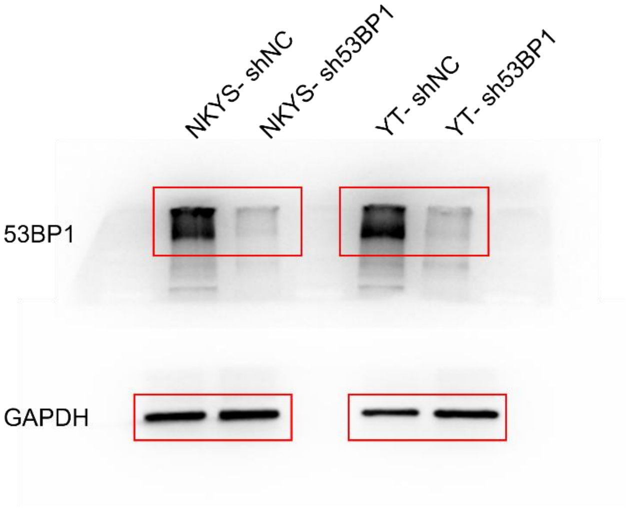

Fig. S5. Images of shNC and sh53BP1 cells .
